# Deqi sensations without cutaneous sensory input: results of an RCT

**DOI:** 10.1186/1472-6882-10-81

**Published:** 2010-12-28

**Authors:** Norbert Salih, Petra I Bäumler, Michael Simang, Dominik Irnich

**Affiliations:** 1 Multidisciplinary Pain Center, Department of Anesthesiology, University of Munich, Munich, Germany; 2 Institute for Medical Information Sciences, Biometry, and Epidemiology, University of Munich, Munich, Germany

## Abstract

**Background:**

Deqi is defined in relation to acupuncture needling as a sensory perception of varying character. In a recently published sham laser validation study, we found that subjects in the verum and the sham laser group experienced deqi sensations. Therefore, we aim to further analyze whether the perceptions reported in the two study arms were distinguishable and whether expectancy effects exhibited considerable impact on our results.

**Methods:**

A detailed re-analysis focusing on deqi sensations was performed from data collected in a previously published placebo-controlled, double-blind, clinical cross-over trial for a sham laser evaluation. Thirty-four healthy volunteers (28 ± 10.7 years; 16 women, 18 men) received two laser acupuncture treatments at three acupuncture points LI4 (hégu), LU7 (liéque), and LR3 (táichong); once by verum laser and once using a sham device containing an inactive laser in randomized order. Outcome measures were frequency, intensity (evaluated by visual analogue scale; VAS), and quality of the subjects' sensations perceived during treatments (assessed with the "acupuncture sensation scale").

**Results:**

Both, verum and the sham laser acupuncture result in similar deqi sensations with regard to frequency (p-value = 0.67), intensity (p-value = 0.71) and quality (p-values between 0.15 - 0.98). In both groups the most frequently used adjectives to describe these perceptions were "spreading", "radiating", "tingling", "tugging", "pulsing", "warm", "dull", and "electric". Sensations reported were consistent with the perception of deqi as previously defined in literature. Subjects' conviction regarding the effectiveness of laser acupuncture or the history of having received acupuncture treatments before did not correlate with the frequency or intensity of sensations reported.

**Conclusions:**

Since deqi sensations, described as sensory perceptions, were elicited without any cutaneous sensory input, we assume that they are a product of non-specific effects from the overall treatment procedure. Expectancy-effects due to previous acupuncture experience and belief in laser acupuncture do not seem to play a major role in elicitation of deqi sensations. Our results give hints that deqi might be a central phenomenon of awareness and consciousness, and that its relevance should be taken into account, even in clinical trials. However, further research is required to understand mechanisms underlying deqi.

## Background

Deqi is defined as a sensation that occurs during positioning of an acupuncture needle during treatment, which can be felt by the patient, the therapist, or both [1-3]. The term "deqi" is derived from two Chinese characters; "得" for "de" which means "arrival" and "氣" for "qi" that can be translated as "life energy". According to the concept of Traditional Chinese Medicine (TCM) a blockage of the natural flow of the qi is seen as the cause of disease. Deqi therefore is supposed to occur when such a blockage is removed and the qi flow is reconstituted [4].

The classical TCM textbook of Huangdi Neijing states that deqi must be felt by the therapist who also needs to concentrate in order to hold it [5]. Later textbooks also refer to patients' sensations [3]. According to both ancient Traditional Chinese and modern text books patients experience deqi very differently and refer to a feeling of soreness, numbness, distension or heaviness around the acupuncture point and/or along the meridians [6,7]. Meanwhile acupuncturists' perception of deqi has been described in terms of a slight pull of the needle downwards into the tissue [4]. There is also evidence to support that the increase of needle adhesion, that has been associated with deqi, is stronger at acupuncture points than at non-acupuncture points [8]. Even greater needle adhesion was reported with bidirectional needle rotation when compared to unidirectional. The needle adhesion has been shown to be due to fasciae winding around the needle both by ultrasound in humans and microscopically in animal models [9].

There are claims that deqi is essential to achieve the intended treatment effects. This issue prompted researchers to conduct trials regarding the physiology underlying or associated with the phenomenon of deqi. Needle stimulation eliciting deqi has e.g. been shown to cause a more intense increase of blood flow in skin and muscle than needle stimulation without deqi [10,11]. Regarding the analgesic effect of acupuncture it is assumed inter alia that needling causes a C-fiber stimulation, which leads to a decrease in pain transmission e.g. via activation of spinal segmental and descending inhibitory systems [12]. Slowly conducting C-and Aδ-fibers in turn are also assumed to play a major role in the perception of deqi, but the complexity of deqi sensations suggests an involvement of a wide range of nerve afferents [7]. This also becomes apparent in brain imaging studies that examine mechanisms that underlie or that are associated with deqi in the central nervous system. Hsieh et al. showed via positron emission tomography, that elicitation of deqi at LI4 resulted in a significant increase of blood flow in the hypothalamus and insula with an extension to the midbrain when compared with minimal or no stimulation after needle insertion at LI4. Off point needling accompanied by the subject's perception of deqi however did not alter cerebral activity in these regions [13]. Hui et al. found that neuronal hemodynamic activity assessed by fMRI following acupuncture of ST36 depends on the kind of needle stimulation used. Attenuated activity can be noted during acupuncture stimulation with elicitation of deqi in limbic and paralimbic structures of cortical and sub cortical regions of the diencephalon, the brainstem and cerebellum. In contrast, hemodynamic activity was mainly increased in the above mentioned limbic brain regions if the needle sensation was accompanied by a sharp pain perception or when a cutaneous, tactile control stimulus was applied [14]. In a recent work Napadow et al. monitored continuously subjects rating of acupuncture sensations during fMRI. They found, that perceptions from needle acupuncture are preferentially processed by the dorsomedial prefrontal cortex when compared to non-penetrating, cutaneous stimulation. The authors conclude that deqi fosters acupuncture analgesia through focusing attention and accentuate the bodily awareness which in turn could enhance antinocipeptive top down mechanisms within the central pain network. There have been some attempts to investigate the quality of deqi sensations. For example, Vincent et al. adapted the McGill Pain Questionnaire creating a scale of 20 adjectives describing possible sensations that patients might feel at the site of needling with a scoring of 0 ("not at all") to 3 ("severely") [15]. Park et al. modified this approach by adding five further sensations [16]. MacPherson and Asghar distinguished the sensations of Park's scale with regard to whether they were associated with deqi or with pain using a rating which was developed by an international group of acupuncture experts. They found that the added adjectives were not included in the deqi cluster. Only seven sensations appeared to be associated with deqi; namely "aching", "dull", "heavy", "numbing", "radiating", "spreading" and "tingling" [6]. Another approach to assess deqi sensations was published by Kong et al. They introduced the "Subjective Acupuncture Sensation Scale" (SASS), which includes only nine descriptors of deqi sensations based on traditional and contemporary literature and one supplementary row for subjects to describe perceptions in their own words. Using this instrument they were able to show significant correlations between the feeling of numbness as well as soreness and the analgesic effect of acupuncture [17]. Later on, a revised version of the SASS the MASS (MGH Acupuncture Sensation Scale) was developed in cooperation with other research groups, which includes two supplementary scales for spreading sensations and anxiety [18].

With regard to the frequency of these sensations, it is known that during needle acupuncture at classical acupuncture points, deqi sensations are elicited in the majority of cases [7,19]. The fact that deqi sensations may unblind volunteers in placebo-controlled trials also adds certain interest to this topic [19]. In the course of validating the Streitberger needle in 34 of 60 cases, an elicitation of deqi was reported in the acupuncture arm, while only 13 subjects treated with the placebo device experienced similar sensations [20]. On the contrary, White et al. did not find an overall difference in needle sensation when reevaluating the placebo needle [21]. The same results apply to a study conducted by Fink and Karst comparing placebo needles to real acupuncture needles [22].

For laser acupuncture, some authors have reported similar deqi sensations that have been described with needle acupuncture [23,24]. In addition fMRI analysis has shown similar cerebral activation patterns during treatment with low intensity laser at acupuncture points as during needle acupuncture [23]. However there have also been reports that volunteers did not perceive deqi during laser acupuncture [25].

Recently our group conducted a clinical trial which provided evidence that sham laser acupuncture can be used as a valid control for laser acupuncture trials. Interestingly, we found that healthy subjects reported deqi sensations not only during acupuncture with an active but also with an inactive laser. The verum and the sham laser could not be distinguished by patients [26].

Given the complexity of acupuncture-dependent effects when using a laser, our data suggested that deqi sensations as subjective perceptions with sensory characteristics could be elicited without a cutaneous sensory input. To verify this assumption we conducted the following more detailed re-analysis of these data.

## Methods

### Study Design

We performed a detailed re-analysis focusing on deqi sensations based on our previously published data collected in a placebo-controlled, double-blind, cross-over clinical trial comparing sham versus verum laser. This study was initially designed to evaluate the sham laser as a placebo device for acupuncture trials. The study was conducted at the Interdisciplinary Pain Center, Department for Anesthesiology, University of Munich. Thirty-four healthy subjects (28 ± 10.7 years; 16 women, 18 men) were recruited through advertisement in student dormitories and hospital staff social rooms. Subjects were randomly allocated into two groups with each group receiving either verum laser or sham laser acupuncture in a cross-over design. Subjects were informed that during one of the treatment episodes a sham laser device would be used. Three acupuncture points were treated during each of the two acupuncture sessions. For each of these six acupuncture point treatments, the subjects' perceptions were assessed regarding quality and intensity of their sensations. Measurements were conducted at the same time of day and at an interval of a minimum of three and a maximum of four days between treatment episodes.

### Randomized allocation and blinding

The randomization list was administrated by an external physician and was not divulged to study practitioners or patients. A series of sealed envelopes containing the treatment assignments were sequentially numbered and used for group allocation. Since neither the study practitioner nor the subjects knew if verum or sham laser acupuncture was applied first, blinding was assured.

### Subjects

Volunteers were at least 18 years old. Exclusion criteria were: local and general contraindications for treatment such as participation in other studies, (laser-) acupuncture treatment in the week prior to or during the examination, taking any medication (except contraceptives), pregnancy, coagulopathies, psychosis or other severe diseases. These criteria were determined both by the examiner and by a questionnaire filled out by the volunteers.

### Ethics

The study protocol was approved by the Ethics Committee of the University of Munich. Written informed consent according to the declaration of Helsinki was given by all volunteers.

### Intervention

Interventions took place at the Interdisciplinary Pain Center, Department for Anesthesiology, University of Munich where a mean room temperature of 22°C is kept. Long hairs at the acupuncture points selected for the treatment were cut with scissors in order to avoid tactile stimuli during treatments.

#### Verum laser acupuncture

Verum laser acupuncture was performed with an infrared (IR) laser of low intensity (Seirin, 3B Scientific GmbH, Hamburg, Germany) and an output power of 22-23 mW. Laser light wavelength was 830 nm (continuous wave) and radiated skin area was 0.78 mm^2 ^resulting in an energy density of 113.0 kJ/cm2. Each treatment episode lasted 45 seconds, and radiation dosage at each point reached 1 kJ. As the control for laser activity, a visual (red LED light of 20 μW) and an acoustic signal was provided. Subjects were treated on the right side at three commonly used acupuncture points: LI4 (hégu), LU7 (liéque), and LR3 (táichong). These were localized without any tactile stimulus and a distance of 5 mm between skin and laser was maintained during treatment. The patient therapist interaction was kept to a minimum. The conversations with the probands were mainly reduced to questions about the sensations they perceived during the treatment sessions.

#### Sham laser acupuncture

**Figure 1:**
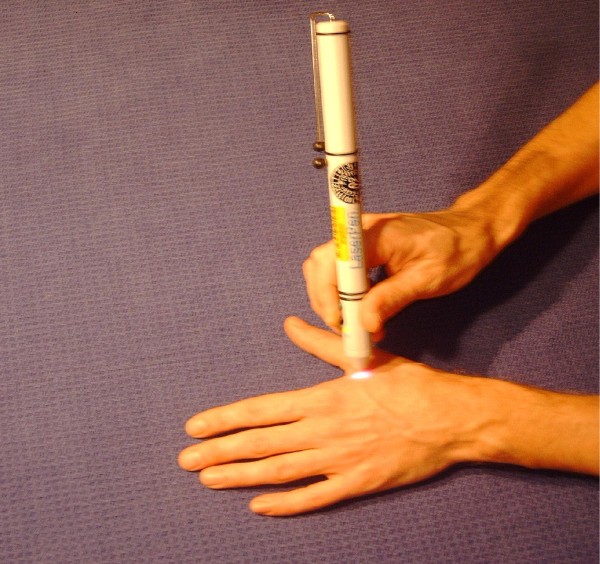
**Sham laser device**. In the device used during sham laser acupuncture, laser irradiation was deactivated by the manufacturer (Seirin, 3B Scientific GmbH, Hamburg, Germany). The visual (red light, approximately 587 nm) and acoustic functions were maintained and therefore double-blinding was achieved.

Sham laser acupuncture followed the same procedure as the verum treatment. In the sham laser device, laser irradiation was deactivated by the manufacturer (Seirin, 3B Scientific GmbH, Hamburg, Germany; Figure 1). The visual (red light, approximately 587 nm) and acoustic functions were maintained in order to make it indistinguishable from the verum device.

### Outcome measures

After treatment of each acupuncture point, we assessed the intensity of skin sensations during or immediately after the treatment on a 10-fold visual analog scale (VAS; with 0 being no sensation and 10 being the strongest imaginable sensation). The quality of these perceptions were evaluated by the "acupuncture sensation scale" [15] which we expanded by two additional adjectives "warm" and "cool". At the end of each treatment episode, the volunteers were asked whether they believed if the laser device was active or inactive. Additional demographic and historic data were collected from the volunteers, including the question whether they believed in the effectiveness of laser acupuncture and if they had received acupuncture treatments in the past.

### Statistical analysis

Thirty-four subjects were analyzed in each study arm and were treated at three different acupuncture points. As a result, the perceptions during 102 point treatments could be analyzed per study group. Statistical analysis was carried out with IBM SPSS statistical software system (SPSS Inc., Chicago, IL; version 17.0). Descriptive data are expressed as mean ± standard deviation. The identification of possible correlations between categorical variables was analyzed by using the chi-square-pearson test for cross-tables. Continuous data (e.g. VAS) were determined with Mann-Whitney-U test. In general a p-value < 0.05 was considered statistically significant.

## Results

In our previously published study we have already shown that subjects were not able to distinguish between verum and sham laser acupuncture. Deqi sensations were reported with equal frequency and with similar intensity in both study groups [26].

### Differentiation of the laser devices

In this more detailed data analysis, we found that the two study groups did neither differ in how often the laser device was identified correctly nor in how often the laser device was assumed to be active. The comparison of the outcome parameters displayed in Table 1 between the first and the second acupuncture session did not result in significant differences. The two laser devices could not be distinguished, regardless of whether subjects were asked after the first treatment session or after they had received both acupuncture treatments with the verum and the sham laser device. Furthermore, no differences were found in the frequency and intensity of reported perceptions at the three different acupuncture points. In both study groups subjects reported to feel deqi sensations more often at LU7 than at LI4 and at LR3 (Table 2).

**Table 1:** Comparison of the verum and the sham laser group, first and second treatment day and impact of expectancy effects due to acupuncture experience and belief in laser acupuncture

	Occurrence of deqi^i)^	Intensity of deqi^ii) ^(± SD)	Laser assumed to be active^iii)^	Correct identifications^iii)^
**verum laser** **(n = 34)**	47/102	2.34 ± 2.34	12/34	12/34
**sham laser (n = 34)**	50/102	2.49 ± 2.36	15/34	17/34
**p-value**	0.67	0.71	0.45	0.21

**day 1** **n = 34**	55/102	2.39 ± 2.45	17/34	15/34
**day 2** **n = 34**	42/102	2.46 ± 2.22	10/34	14/34
**p-value**	0.07	0.69	0.18	0.84

**acupuncture-experienced n = 17**	53/102	3.24 ± 2.65	14/34	12/34
**acupuncture-naïve n = 17**	44/102	1.43 ± 1.37	13/34	17/34
**p-value**	0.21	0.0002	0.97	0.14

**convinced n = 14**	44	2.23 ± 1.80	13/28	12/28
**not convinced n = 20**	53/120	2.57 ± 2.72	14/40	17/40
**p-value**	0.25	0.72	0.30	0.91

Statistical analysis was performed by using the chi-square-test according to Pearson for occurrence of deqi sensations, for how often the laser device was assumed to be active and for the number of correct identifications. The Mann-Whitney-U test was used to compare the intensity of deqi sensations (evaluated by VAS). Subjects were treated at two treatment sessions at three different acupuncture points. Therefore, occurrence of deqi sensations is depicted relative to the number of point treatments calculated as *n *times six. Subjects were asked whether they considered the laser device active or inactive after each treatment session, hence correct identifications and the estimations that the laser was active apply to the total number of treatments calculated as *n *times 2.

**Table 2:** Comparison between the two study arms regarding occurrence and intensity of perceived sensations at the three different acupuncture points

	Occurrence of deqi		
	LI4	LU7	LR3
**verum laser n = 47**	14	18	15
**sham laser n = 50**	11	24	15
**p-value**	0.151	0.268	0.607

	**Intensity (± SD) of deqi**		
	**LI4**	**LU7**	**LR3**

**verum laser n = 47**	2.21 ± 2.15	2.67 ± 2.39	2.08 ± 2.57
**sham laser n = 50**	2.67 ± 2.37	2.26 ± 1.99	2.73 ± 2.96
**p-value**	0.61	0.55	0.76

Statistical analysis was performed by using the chi-square-test according to Pearson for occurrence of deqi sensations and by the Mann-Whitney-U test for the intensity of these perceptions (evaluated by VAS, 0-10).

### Quality of perceived sensations

**Figure 2:**
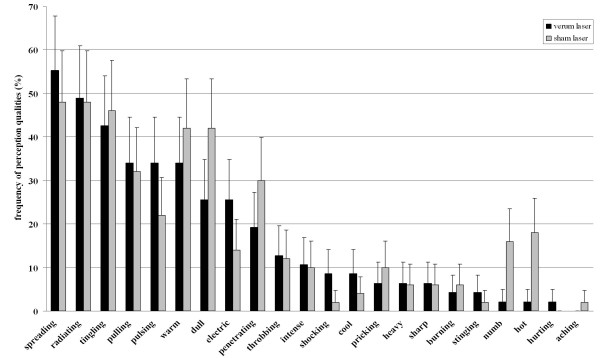
**Comparison of frequencies with which adjectives were used to describe perceptions during acupuncture treatment with the verum laser and the sham laser**. Relative frequencies were calculated upon the number of subjects reporting perceptions within each study arm (verum laser group n = 47, sham laser group n = 50). We included errors bars representing the 95% confidence interval for this binary distribution. For more details on data refer to Table three.

Frequencies of adjectives used to characterize perceptions during acupuncture treatments are displayed in Figure 2 and Table 3 for both study groups. Both verum (VL) and sham laser acupuncture (SL) evoked the most common sensations described as "spreading" (VL 55%; SL 48), "radiating" (VL 49%; SL 48%), "tingling" (VL 43%; SL46%), "pulling" (VL 34%; SL 32%), "pulsing" (VL 34%; SL 22%), "warm" (VL 34%; SL 42%), "dull" (VL 26%; SL 42%), "electric" (VL 26%; SL 14%) and "penetrating" (VL 19%; SL 30%). Descriptively the two study groups differed in how often these qualities were named in order to characterize perceptions. During verum laser acupuncture, the sensations of "spreading", "pulsing" and "electric" were reported more frequently than in the sham laser group. Qualities reported more often in the sham laser than in the verum laser group were "warm", "dull", and "penetrating". These discrepancies, however, lacked statistical significance (p-values between 0.15 - 0.98; chi-square-pearson). The study was not sufficiently powered to allow for meaningful statistical comparison between the study groups with regard to the reports of the following adjectives: "throbbing", "intense", "shocking", "cool", "pricking", "heavy", "sharp", "burning", "stinging", "numbing", "hot", "hurting", "aching" (Table 3).

**Table 3:** Absolute frequency of adjective used to describe perceptions during the acupuncture treatments

	verum laser (n = 47)	sham laser (n = 50)	p-values
**spreading**	26	24	0.47
**radiating**	23	24	0.93
**tingling**	20	23	0.98
**pulling**	16	16	0.83
**pulsing**	16	11	0.19
**warm**	16	21	0.73
**dull**	12	21	0.32
**electric**	12	7	0.15
**penetrating**	9	15	0.46
**throbbing**	6	6	-
**intense**	5	5	-
**shocking**	4	1	-
**cool**	4	2	-
**pricking**	3	5	-
**heavy**	3	3	-
**sharp**	3	3	-
**burning**	2	3	-
**stinging**	2	1	-
**numbing**	1	8	-
**hot**	1	9	-
**hurting**	1	0	-
**aching**	0	1	-

Relative frequencies were calculated upon the number of subjects reporting perceptions within each study arm (verum laser group n = 47, sham laser group n = 50). Statistical comparison by chi-square-test according to Pearson did not reveal significant differences between the two study groups (p-values between 0.15 - 0.98).

### Impact of previous acupuncture experience

Half of the subjects had received acupuncture treatments in the past (n = 17). As already reported in our previous publication [26], subjects having experienced acupuncture before noted deqi sensations with similar frequency but with greater intensity (p < 0,001). Acupuncture experienced subjects were not able to identify the verum laser more frequently when compared to acupuncture naïve subjects. Furthermore, our more detailed data analysis shows that the two subgroups did not differ in their overall ability to identify the verum or the sham laser, nor in how often the laser devices were assumed to be active (Table 1).

### Impact of belief in laser acupuncture

With further regard to expectancy effects, we analyzed the influence of belief in laser acupuncture. The study population included 14 subjects with the conviction that laser acupuncture is an effective treatment and 20 subjects who did not believe in the effectiveness of laser acupuncture. These two subgroups reported deqi sensations with similar frequency and intensity. Subjects with the belief in the efficacy of laser acupuncture endorsed that the laser device was active 13 times out of 28 treatment sessions, while subjects without belief in laser acupuncture presumed 14 times out of 40 treatment sessions that the laser was turned on (p = 0.30; chi-square-pearson). Whether subjects were convinced of the effectiveness of laser acupuncture or not also did not result in significant discrepancies regarding the number of correct identifications of the laser devices as active or inactive, respectively (Table 1).

## Discussion

In the course of evaluating a sham laser device as a valid placebo for acupuncture trials, we found that healthy subjects reported deqi sensations not only during treatments with an active but also with an inactive laser [26]. We therefore conjectured that deqi sensations as perceptions with sensory characteristics can be elicited without cutaneous sensory input. With the more detailed data analysis, we were able to collect evidence supporting this hypothesis.

In both groups subjects most frequently described what they felt with the following adjectives: "spreading", "radiating", "tingling", "pulling", "pulsing", "warm", "dull", "electric" and "penetrating" (Figure 2). These correlate extensively with qualities used in literature to characterize the perception of deqi [6,16]. However the adjective "heavy", which is consistently named in the cluster associated with deqi was only used by 6% of all subjects reporting perceptions in both study arms. This might be due to the fact that here "heavy" was translated by "heftig" having the sense of "intense" instead of "schwer" which has the sense of "weighty" as it is derived from the validated German version of the McGill Pain Questionnaire [27]. Also the adjective "penetrating" was used more frequently than in other acupuncture trials focusing on deqi sensations. This may be the result of its German translation as "eindringend", which does not necessarily describe the perception of piercing the skin but suggests more an infiltrating or streaming quality. The remaining qualities listed in the questionnaire are more often associated with pain and were less frequently used to characterize perceptions during treatment with both the verum laser and the sham laser. Therefore it can be assumed that the sensations reported in our study are of the same nature as the feeling of deqi as it is defined in ancient Chinese text books and in modern studies that have evaluated sensations during acupuncture treatments.

As shown in Table 1, 2 and 3 and Figure 2 verum and sham laser acupuncture was surprisingly experienced in a similar way; also irrespective of whether the subjects were assessed after the first treatment session or after they had received both acupuncture treatments with the verum and the sham laser device. Subjects did not identify one of the laser devices more often correctly than the other and did believe them to be active with the same frequency. Thus one can conclude that, besides the deqi sensations occurring with equal frequency and similar intensity, there were no other differences between the two laser devices that made them distinguishable. In order to achieve more detailed information we checked for whether there were any laser specific effects on the quality of the perceptions reported. However, the comparison of the two study groups did not show any significant differences between the frequencies with which adjectives describing deqi sensations were used (Table 3). Taken together, our results do not display any relevant differences between perceptions reported during treatment with the active and the inactive laser, with regard to frequency, intensity, or quality.

Therefore, we conclude that deqi sensations were not caused by laser light specific effects. There are several conceivable reasons for our observations. First of all, the case numbers were calculated for the purpose of evaluating the efficacy of the sham laser as a placebo device. This appears to not be sufficient to distinguish differences between all of the various qualities of the perceptions evoked by verum and sham laser acupuncture. Therefore we conclude that further investigations based on a larger study population would be needed to confirm or reject the clinical relevance of the reported trend towards showing a difference in the quality of the deqi sensations between the study groups.

Regarding the technical aspects of the treatments, it must be noted that we used a rather weak laser and applied a power intensity of only 2.9 W/cm^2^. The recommended dose, however, varies greatly depending on the author [23,24,28]. We chose the laser parameters according to our experience and based on an approach described by Sommer et al., which postulates that the relationship between laser energy and cell activity follows the Arndt-Schultz-rule [29]. This rule states that high power intensities cause a repression of cell activity or even cell damage at higher dosage. The wavelength of 830 nm provides an optimal penetration depth for the laser light through the skin analog to the Kubelka-Munk-theory [29]. Hence we do not believe that the technical parameters led to diminished function of the verum laser. Nonetheless, further evaluation of the dependence between deqi sensations evoked by laser acupuncture and different laser parameters might provide useful information.

Taken together, no obvious methodological factors regarding our conclusion that deqi sensations were elicited by laser light independent mechanisms could be found. Therefore it needs to be questioned what factors may have led to the sensations reported.

Firstly, we must consider that the sham laser device might not be totally inert. Both the verum and the sham laser emit LED red light of the same wavelength, which could have had a physiological effect leading to the reported perceptions. There is evidence given by Stelian et al. that red light has a therapeutic effect on osteoarthritic pain when compared to invisible infrared or placebo light emitters [30]. However, in this trial, an incandescent bulb emitting coherent narrow-band light was applied for 15 minutes, and twice a day for 10 days. In contrast we used incoherent light (LED) with an output power of only 20 μW and the treatment duration was 45 s each. Therefore, it seems unlikely that the red light could cause a significant physiologically relevant stimulus. One can also rule out that tactile stimuli caused the perceptions described by subjects since the acupuncture points were localized without any skin contact, and during treatment, a distance between skin and laser of 5 mm was maintained. Taken together we conclude that in our study the perceived deqi sensations are not provoked by mechanisms analog to the classical definition of sensory perception.

Moreover, non-specific effects, for example, due to expectancy, awareness, focusing the attention on the acupuncture points, or general treatment settings may play an important role in the elicitation of deqi. With regard to subjects' expectancy, interestingly we found that the subjects' conviction of whether laser acupuncture was an effective treatment or not did not correlate with the frequency or the intensity (Table 1) of deqi sensations reported. Subjects believing in laser acupuncture did not consider the laser devices more often as active nor could they distinguish between the active and the inactive laser.

The same subgroup analysis was performed regarding the influence of previously having experienced acupuncture treatments. Patients who had previously received acupuncture reported deqi sensations with higher intensities than those who had no experience with acupuncture. Regarding the other outcome measures, there were no differences between the two subgroups (Table 1). From these additional results, one can draw the conclusion that expectancy whether due to belief or to previous experience, has no influence on whether deqi sensations are perceived or not. The fact that deqi sensations seem to be more intense for subjects that had received acupuncture before might be related to increased familiarity with perceiving deqi. Retrospectively, it would have been interesting to ask the question in the interviews if subjects having received acupuncture before also had experienced deqi in these previous treatments.

A fact that also must be considered is that patients were told to pay attention to what they felt during treatments, hence reinforcing the expectation that they would feel something.

Therefore, the question arises which mechanisms - whether of a psychological or a physical nature - underlie our observation that neither frequency, intensity nor quality of sensations reported depended on the activation status of the laser device. To answer this question further investigation is needed, but one can speculate about possible mechanisms. When one looks at what actually happens during an acupuncture treatment - in our case during laser acupuncture - we find a unique physician patient interaction characterized by attention particularly paid on the acupuncture point. By concentrating on a certain body area one could become aware of spontaneous activity of motoneurons or peripheral sensory afferents such as for example muscle or skin sympathetic nerve activity [31]. On the one hand, there is the patient's expectancy that he or she might feel something during the treatment as aforesaid. But on the other hand, there is the therapists' expectation. As taught during acupuncture schooling, the acupuncturist aims to reconstitute the flow of qi and open the energy flow blockage. This is to say, therapists have a particularly defined intention. Especially in the context of various treatment methods approaches in the field of complementary and alternative medicine, the term "healing intention" is commonly used. It describes the general benevolent desire for another human, the intention to improve the patient's health situation, or simply, a state in which life is enhanced [32]. There is some evidence for the effectiveness of such healing intention as applied in distant healing, Reki, and others in several medical conditions [33,34] as well as for observed effects on living systems in vitro [35-37]. In order to explain these phenomena, current literature postulates the existence of a biofield or bio-electromagnetic interaction models [38,39]. Albeit, it has been reported that such mental intention can cause perceptions in subjects to whom they are directed [40] providing one possible explanation for why sensory perceptions were reported in our study, even though no cutaneous sensory stimulus was applied. However, further research is required to define the mechanism underlying the deqi phenomena.

## Conclusion

Taken together the presented results demonstrate that the verum and the sham laser result in similar deqi sensations with regard to frequency, intensity and quality. Expectancy-effects due to previous acupuncture experience and belief in laser acupuncture do not seem to play a major role in elicitation of deqi sensations.

Since these sensory perceptions were elicited without any cutaneous sensory input, we assume that they are a product of non-specific effects from the overall treatment procedure. Our results give hints that deqi might be a central phenomenon of awareness and consciousness and that its relevance should be taken into account, even in clinical trials.

However, further research is required to fully understand mechanisms underlying this phenomenon.

## Competing interests

The authors declare that they have no competing interests.

## Authors' contributions

NS took part in planning the study, performed the experiments, and provided advice for the manuscript work.

PIB performed the data management analysis and did write the manuscript.

MS performed the statistical analysis.

DI is in authority for study planning as well as execution and assisted the manuscript work.

All authors read and approved the final manuscript.

## Pre-publication history

The pre-publication history for this paper can be accessed here:

http://www.biomedcentral.com/1472-6882/10/81/prepub
